# Survival analysis of patients with COVID-19 admitted at six hospitals in Uganda in 2021: a cohort study

**DOI:** 10.1186/s13690-022-00991-3

**Published:** 2022-11-15

**Authors:** Asad Muyinda, Prossie M. Ingabire, Susan Nakireka, Criscent Tumuhaise, Edith Namulema, Felix Bongomin, Agnes Napyo, Quraish Sserwanja, Rozen Ainembabazi, Ronald Olum, Ritah Nantale, Phillip Akunguru, Derrick Nomujuni, William Olwit, Milton W. Musaba, Bridget Namubiru, Pamela Aol, Peter A. Babigumira, Ian Munabi, Sarah Kiguli, David Mukunya

**Affiliations:** 1 Department of Medicine, Jinja Regional Referral Hospital, Jinja, Uganda; 2 Department of Medicine, Nsambya Hospital, Kampala, Uganda; 3 Department of medicine, Mengo Hospital, Kampala, Uganda; 4 Department of Medicine and Dentistry, Uganda Christian University, Kampala, Uganda; 5 Department of Medicine, Our Lady Health of the Sick, Nkozi Hospital, Nkozi, Uganda; 6 Covid Task Force Institution, Mengo Hospital, Kampala, Uganda; 7 Department of Medical Microbiology, Gulu University, Gulu, Uganda; 8 Department of Community and Public Health, Busitema Universiy, Tororo, Uganda; 9 Department of Programmes, GOAL, Arkaweet Block 65 House No, 227 Khartoum, Sudan; 10 Department of Medicine, Jaro Hospital, Wakiso, Uganda; 11 Department of Nursing, Busitema University, Tororo, Uganda; 12 Department of Medicine, Moroto Hospital, Moroto, Uganda; 13 Department of Medicine, Mulago Hospital, Kampala, Uganda; 14 Department of Medicine, Case Hospital, Kampala, Uganda; 15 Department of Obstetrics and Gynaecology, Mbale Regional Referral and Teaching Hospital, Mbale, Uganda; 16 Department of Obstetrics and Gynaecology, Busitema University, Tororo, Uganda; 17 Department of Pediatrics, Lacor Hospital, Gulu, Uganda; 18 Department of Global Health Security, Ministry of Health, Kampala, Uganda; 19 Department of Anatomy, Makerere University, Kampala, Uganda; 20 Department of Pediatrics and Child Health, Makerere University, Kampala, Uganda

**Keywords:** COVID-19, Survival, Uganda

## Abstract

**Background:**

Assessing factors associated with mortality among COVID-19 patients could guide in developing context relevant interventions to mitigate the risk. The study aimed to describe mortality and associated factors among COVID-19 patients admitted at six health facilities in Uganda.

**Methods:**

We reviewed medical records of patients admitted with COVID-19 between January 1st 2021 and December 31st 2021 in six hospitals in Uganda. Using Stata version 17.0, Kaplan Meier and Cox regression analyses were performed to describe the time to death and estimate associations between various exposures and time to death. Finally, accelerated failure time (AFT) models with a lognormal distribution were used to estimate corresponding survival time ratios.

**Results:**

Out of the 1040 study participants, 234 (22.5%: 95%CI 12.9 to 36.2%) died. The mortality rate was 30.7 deaths per 1000 person days, 95% CI (26.9 to 35.0). The median survival time was 33 days, IQR (9–82). Factors associated with time to COVID-19 death included; age ≥ 60 years [adjusted hazard ratio (aHR) = 2.4, 95% CI: [1.7, 3.4]], having malaria test at admission [aHR = 2.0, 95% CI:[1.0, 3.9]], a COVID-19 severity score of severe/critical [aHR = 6.7, 95% CI:[1.5, 29.1]] and admission to a public hospital [aHR = 0.4, 95% CI:[0.3, 0.6]]. The survival time of patients aged 60 years or more is estimated to be 63% shorter than that of patients aged less than 60 years [adjusted time ratio (aTR) 0.37, 95% CI 0.24, 0.56]. The survival time of patients admitted in public hospitals was 2.5 times that of patients admitted in private hospitals [aTR 2.5 to 95%CI 1.6, 3.9]. Finally, patients with a severe or critical COVID-19 severity score had 87% shorter survival time than those with a mild score [aTR 0.13, 95% CI 0.03, 0.56].

**Conclusion:**

In-hospital mortality among COVID-19 patients was high. Factors associated with shorter survival; age ≥ 60 years, a COVID-19 severity score of severe or critical, and having malaria at admission. We therefore recommend close monitoring of COVID-19 patients that are elderly and also screening for malaria in COVID-19 admitted patients.

**Supplementary Information:**

The online version contains supplementary material available at 10.1186/s13690-022-00991-3.

## Introduction

Coronavirus disease-2019 (COVID-19) is caused by a novel coronavirus called severe acute respiratory syndrome coronavirus-2 (SARS-CoV-2) and is associated with high morbidity and mortality [1]. On 11th March 2020, the World Health Organization (WHO) declared COVID-19 a pandemic, after 118,000 cases and 4291 deaths were reported in 114 countries [2]. As of 15th July 2022, over 565.6 million confirmed cases and more than 6.4 million deaths have been reported worldwide [1]. In Uganda, the first case of COVID-19 was reported on 21st March 2020, and as of 15th July 2022 over 168, 000 confirmed cases and over 3600 deaths had been reported [3, 4].

The clinical presentations of COVID-19 range from an asymptomatic infection, mild disease to severe illness and death [5]. Majority of the patients have asymptomatic to mild COVID-19 disease and about 15% of the COVID-19 patients suffered severe illness which presented as need for hospitalization, supplemental oxygen or mechanical ventilation [6, 7]. A number of studies conducted in mostly high income countries have revealed that the factors associated with severe COVID-19 illness and mortality are older age, male gender, and having comorbidities such as hypertension, diabetes, cardiovascular diseases and respiratory diseases [8–21]. Low-income countries could have unique factors associated with mortality. Some of the deaths could be attributed to health system related factors. COVID-19 patients overwhelmed health care facilities that were already overloaded by patients with HIV/AIDS, pneumonia, malaria, and tuberculosis and patients who need surgery [22, 23]. The low health system capacity ranging from low physician density especially anesthesiologists, hospital beds, personal protective equipment, diagnostic capacity and critical care services could increase COVID-19 morbidity and mortality rates [22, 24]. Additionally, the raised health care costs such as the price of some antiviral drugs, scarcity of personal protective equipment such as N95 face masks, lack of ventilators and inadequate intensive care unit beds have led to deficiencies in provision of care [25]. Secondary and primary health care facilities especially those in rural settings experience shortages in health work force, general equipment and medicine which exposes weakness in the delivery of care [26].

Studies done in Uganda on predictors of in hospital mortality among COVID-19 patients have been mostly single center studies, in urban health facilities and with low numbers [14, 17] and may therefore not be representative of the populations in the rural settings or entire country. Identifying the factors associated with the incidence of mortality among COVID-19 patients in both urban and rural settings could provide a better understanding of the impact of the disease and is important in the formulation of effective preventive and treatment measures. Therefore, in this study, we described in hospital mortality and associated factors among COVID-19 patients admitted at six health facilities in Uganda.

## Materials and methods

### Study design

We conducted a retrospective cohort study between January 2021 and December 2021.

### Study setting

This study was carried out at six hospitals treating COVID-19 patients in Uganda. Two public hospitals (Moroto regional referral hospital and Jinja regional referral hospital), one private hospital; Jaro hospital and three faith-based hospitals (Mengo hospital, Nsambya hospital and Nkozi hospital) were included.

#### Participating health facilities

Mengo hospital is a church-based private not for profit, oldest hospital in the country, having been established by missionaries in 1897 and is owned by the Church of Uganda. Mengo has a bed capacity of over 300 beds. The Mengo CTU is a 48-bed unit accredited by the MOH to provide treatment for moderate and severe cases of COVID-19. The hospital started as a holding centre for COVID-19 in August 2020, actual treatment started in January 2021. Treatment of COVID-19 is at a fee. Nkozi Hospital is a Private Not for Profit (PNFP) Hospital owned by Kampala Archidiocese. It was founded by the White Sisters of Our Lady of Africa from the Netherlands in 1942 in Mawokota County South, off the Kampala-Masaka Road. Nkozi hospital operates 24 hours daily and offers curative, preventive, promotive and referral services both at the static and in outreaches. It has both inpatient and outpatient services. It has a 100-bed capacity. Treatment of COVID-19 is at a fee.

Nsambya hospital is a private not for profit hospital located in the southern part of the capital city Kampala, about 3 km from the city center. Nsambya has approximately 400 bed capacity hospital. Treatment of COVID-19 is at a fee. Moroto Hospital is a public hospital funded by the Uganda Ministry of Health in Moroto district. Moroto Hospital has a 172-bed capacity; the COVID-19 treatment unit has a capacity of 50 beds. Treatment of COVID-19 is free of charge.

Jinja regional referral hospital is located in Jinja district, approximately 84 km East of the capital city, Kampala. It has a bed capacity of 600 and serves as a referral hospital for 10 districts. Treatment of COVID-19 is free of charge. Jaro hospital is a private for profit hospital located in Wakiso district with approximately 30-bed capacity. Treatment of COVID-19 is at a fee.

### Study sample

We included data for all patients with a polymerase chain reaction (PCR) or rapid diagnostic test (RDT) or radiological confirmation of COVID-19 among patients hospitalized in the six hospitals above, from January 2021 to December 2021. We expected to recruit at least 1000 participants and this would provide absolute precision ranging from 0.9 to 3% for mortality estimates ranging from of 2 to 50%, which we deemed adequate.

### Variables

The outcome variable was time to all cause in-hospital death among patients admitted with COVID-19. The independent variables included socio demographic factors, clinical factors and health system related factors. Independent Clinicians and trained research assistants extracted data from the patient files about demographics and the health system related factors. COVID-19 severity score was defined as:

Mild: Normal Saturation, no need of intravenous treatment.

Moderate: Normal Saturation > =94%, but in need of intravenous medication.

Severe/Critical: Saturation < 94%, and in need of oxygen therapy.

### Data management

Trained research assistants, who were mostly COVID-19 treatment unit nurses, collected the data using a data entry forms designed and administered using KoBo Toolbox (Cambridge, Massachusetts, USA). KoBo Toolbox is open-source software developed by the Harvard Humanitarian Initiative with support from United Nations agencies, CISCO, and partners to support data management by researchers and humanitarian organizations (https://www.kobotoolbox.org/). All completed questionnaires were uploaded onto KoBo Toolbox servers. These servers are secure and encrypted with strong safeguards and protection against data loss. Data was then exported into Microsoft Excel and Stata 17.0 for cleaning, coding, and analysis. Data cleaning included crosschecking data for implausible values, recoding missing observations, checking source documents for missing observations of outcome data among others.

### Statistical analysis

Data were analyzed using Stata version 17.0 (StataCorp LLC, College Station, Texas, United States of America). We summarized continuous variables using means with standard deviations or medians with interquartile ranges and categorical variables using their frequencies and percentages. We drew bar graphs and upset plots (https://gehlenborglab.shinyapps.io/upsetr/) to graphically summarize our data. Fisher’s exact tests were used for comparison of categorical variables.

Mortality was defined as the proportion of COVID-19 patients admitted at the study hospitals between January 2021 and December 2021 who died. We defined mortality rate as the ratio of deaths to the total person time under observation in days. Time to death was calculated as the number of days from admission to death. The total person time contributed by each participant was the number of days from admission to death, discharge, or referral. Kaplan-Meier curves were used to graphically describe survival across selected socio-demographic and clinical characteristics.

Cox proportional hazard models were used to estimate hazard ratios and their 95% confidence intervals between selected exposures from literature and time to death. We calculated a concordance index to check the predictions of our model using Stata’s *estat concordance* test, and this showed a high Harrell’s C value (0.78), signifying that our model performed well. We then tested the proportional hazards assumption using the proportional hazard test in Stata (*estat phtest, detail*). This suggested that two variables violated the assumption: 1) History of HIV infection and 2) COVID-19 severity score. Consequently, we also generated log-log plots to visually assess violation of the proportional hazards assumption. We used Stata’s *stphplot, by (var)* command to draw the log-log plots. As such we also constructed accelerated failure time (AFT) models. To select the appropriate model, we compared the four most common distributions: Weibull, Exponential, Log-Normal, and Log-Logistic and chose the Log-Normal distribution as it had the lowest AIC and BIC values. Finally, we drew Cox-Snell residual plots to access the AFT model adequacy. All variables included in the final models were tested for multicolinearity, but no two variables were strongly collinear (variance inflation factor greater than 5).

## Results

### Participant characteristics

A total of 1040 COVID-19 participants were included in this study. The median age of participants was 52 years, interquartile range (IQR) 34 to 67 years. Slightly more than half of the participants were female, 555/1038 (53.5%). The median (IQR) time from symptom onset to admission was 6 days (4 to 10). The rest of the study characteristics are shown in Table 1 below.

**Table 1:** Participant characteristics of COVID-19 patients admitted in six hospitals in Uganda in 2021

Variables	Alive (*n* = 806)	Dead (*n* = 234)	Total (1040)	*P* value
**Participant Age**				< 0.001
< 18	59 (7.32%)	0 (0.00%)	59 (5.67%)	
18 to < 50	364 (45.16%)	46 (19.66%)	410 (39.42%)	
50-max	382 (47.39%)	188 (80.34%)	570 (54.81%)	
**Sex**				0.602
Female	434 (53.85%)	121 (51.71%)	555 (53.37%)	
Male	371 (46.03%)	112 (47.86%)	483 (46.44%)	
**Study Center**				< 0.001
PNFP	386 (47.89%)	146 (62.39%)	532 (51.15%)	
Public Hospital	407 (50.50%)	86 (36.75%)	493 (47.40%)	
Private Hospital	13 (1.61%)	2 (0.85%)	15 (1.44%)	
**Admitted on weekend**				0.445
No	603 (74.81%)	169 (72.22%)	772 (74.23%)	
Yes	203 (25.19%)	65 (27.78%)	268 (25.77%)	
**Nationality**				0.403
Ugandan	793 (98.39%)	228 (97.44%)	1021 (98.17%)	
Others	13 (1.61%)	6 (2.56%)	19 (1.83%)	
**Marital Status**				0.001
Married/Cohabiting	564 (69.98%)	161 (68.80%)	725 (69.71%)	
Single/Divorced	165 (20.47%)	21 (8.97%)	186 (17.88%)	
**COVID vaccinated**				0.077
No	800 (99.26%)	229 (97.86%)	1029 (98.94%)	
Yes	6 (0.74%)	5 (2.14%)	11 (1.06%)	
**Highest Level of Education**				0.639
None	75 (9.31%)	11 (4.70%)	86 (8.27%)	
Primary	67 (8.31%)	11 (4.70%)	78 (7.50%)	
Secondary	94 (11.66%)	10 (4.27%)	104 (10.00%)	
Tertiary	202 (25.06%)	22 (9.40%)	224 (21.54%)	
**HIV**				0.229
No	781 (96.90%)	223 (95.30%)	1004 (96.54%)	
Yes	25 (3.10%)	11 (4.70%)	36 (3.46%)	
**Hypertension**				< 0.001
No	607 (75.31%)	140 (59.83%)	747 (71.83%)	
Yes	199 (24.69%)	94 (40.17%)	293 (28.17%)	
**Diabetes Mellitus**				< 0.001
No	703 (87.22%)	172 (73.50%)	875 (84.13%)	
Yes	103 (12.78%)	62 (26.50%)	165 (15.87%)	
**COPD**				0.999
No	805 (99.88%)	234 (100.00%)	1039 (99.90%)	
Yes	1 (0.12%)	0 (0.00%)	1 (0.10%)	
**Tuberculosis**				0.999
No	786 (97.52%)	228 (97.44%)	1014 (97.50%)	
Yes	20 (2.48%)	6 (2.56%)	26 (2.50%)	
**Malaria**				0.266
No	748 (92.80%)	212 (90.60%)	960 (92.31%)	
Yes	58 (7.20%)	22 (9.40%)	80 (7.69%)	
**Covid-19 severity score at admission**				< 0.001
Mild	69 (8.56%)	5 (2.14%)	74 (7.12%)	
Moderate	402 (49.88%)	38 (16.24%)	440 (42.31%)	
Severe	326 (40.45%)	158 (67.52%)	484 (46.54%)	
Critical	7 (0.87%)	33 (14.10%)	40 (3.85%)	

### Clinical characteristics

**Fig. 1:**
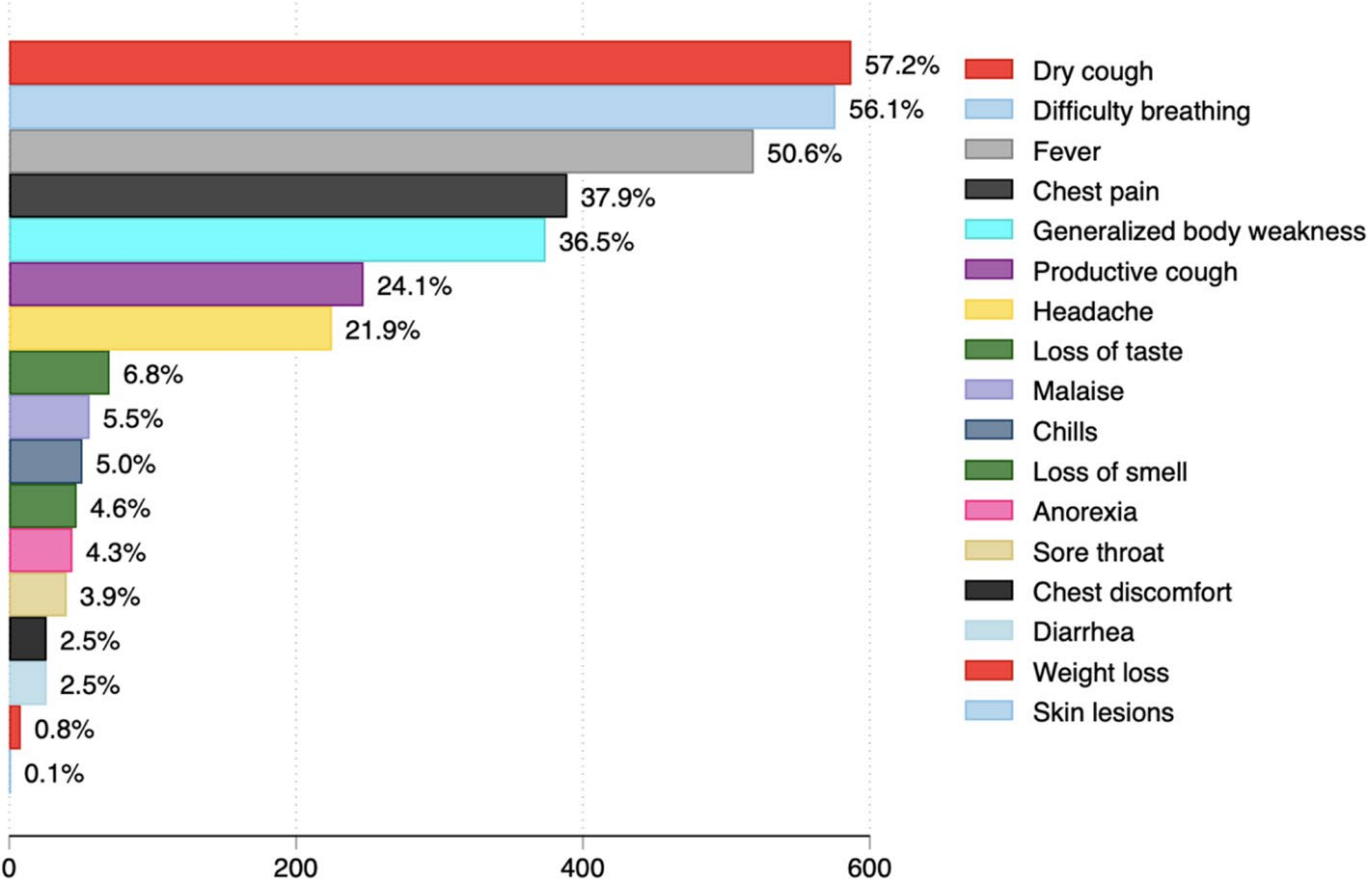
Symptoms experienced by COVID-19 patients hospitalized in selected hospitals in Uganda in 2021

**Fig. 2:**
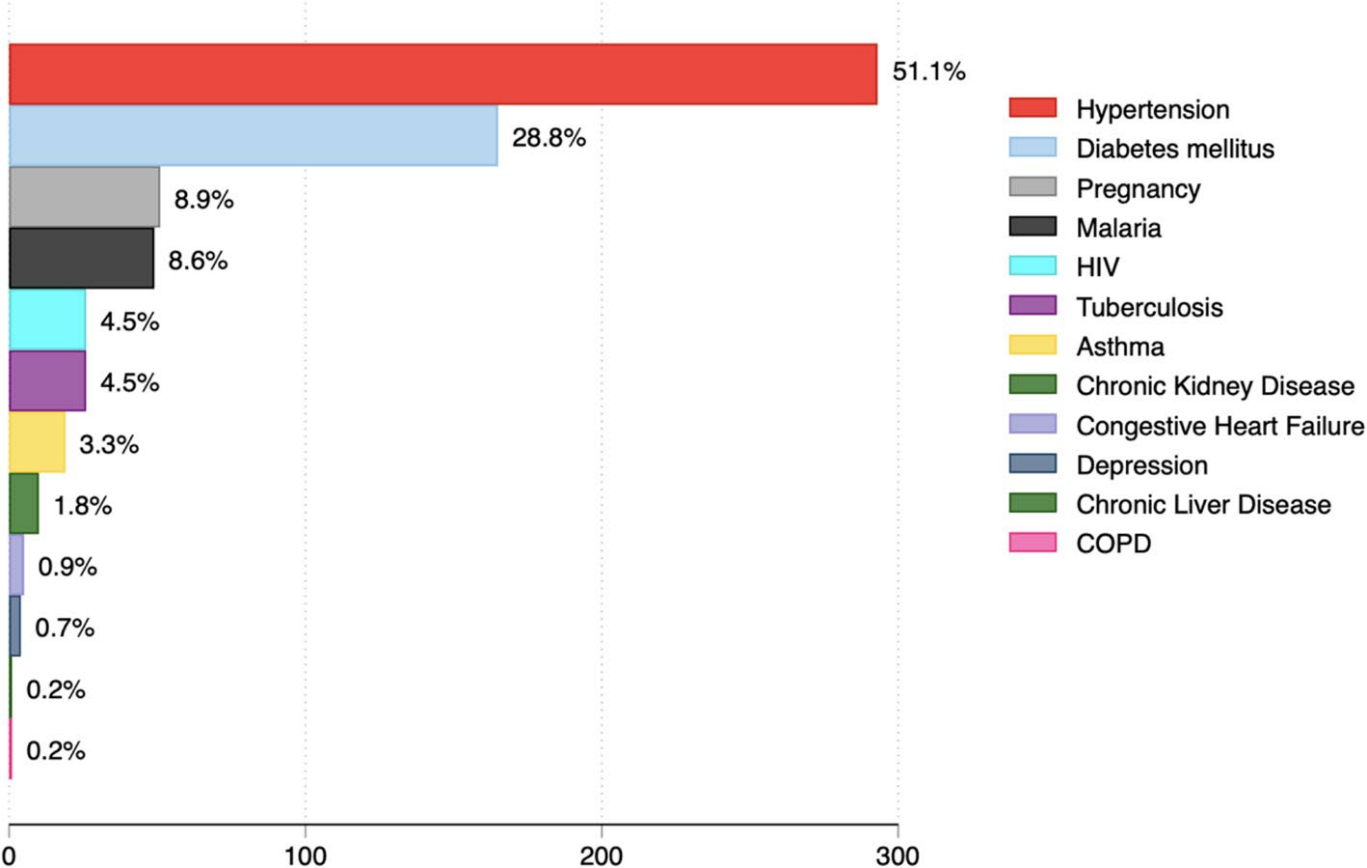
Comorbidities of COVID-19 patients hospitalized in selected hospitals in Uganda in 2021

**Fig. 3:**
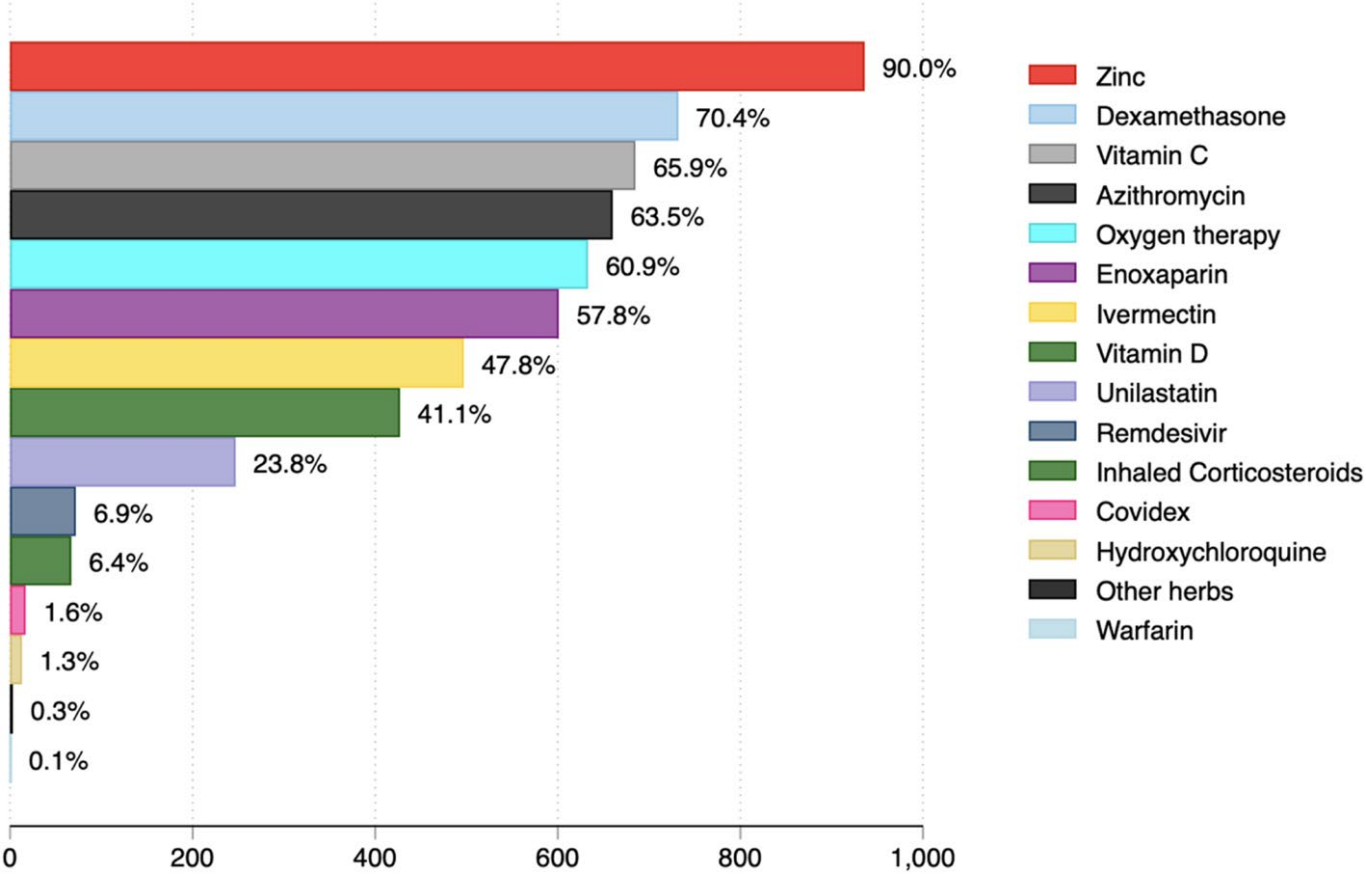
Medications given to COVID-19 patients hospitalized in selected hospitals in Uganda in 2021

**Fig. 4:**
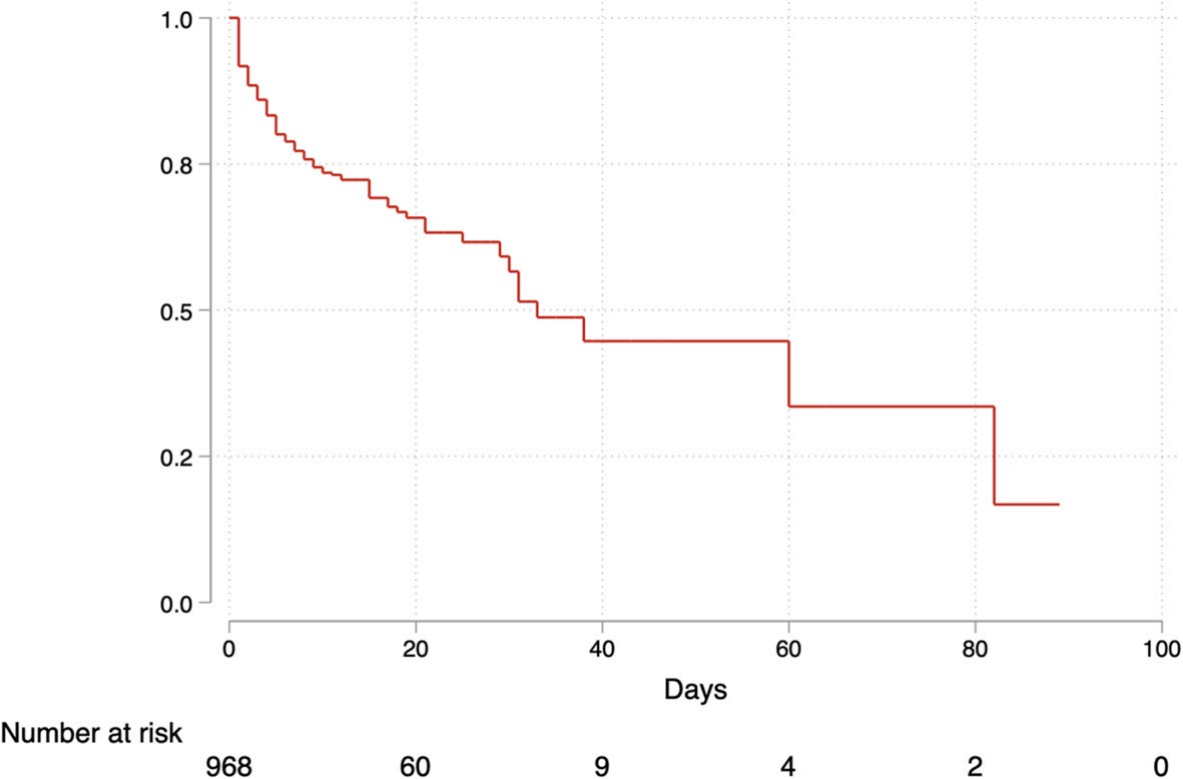
Survival among COVID-19 patients hospitalized in selected hospitals in Uganda in 2021

**Fig. 5:**
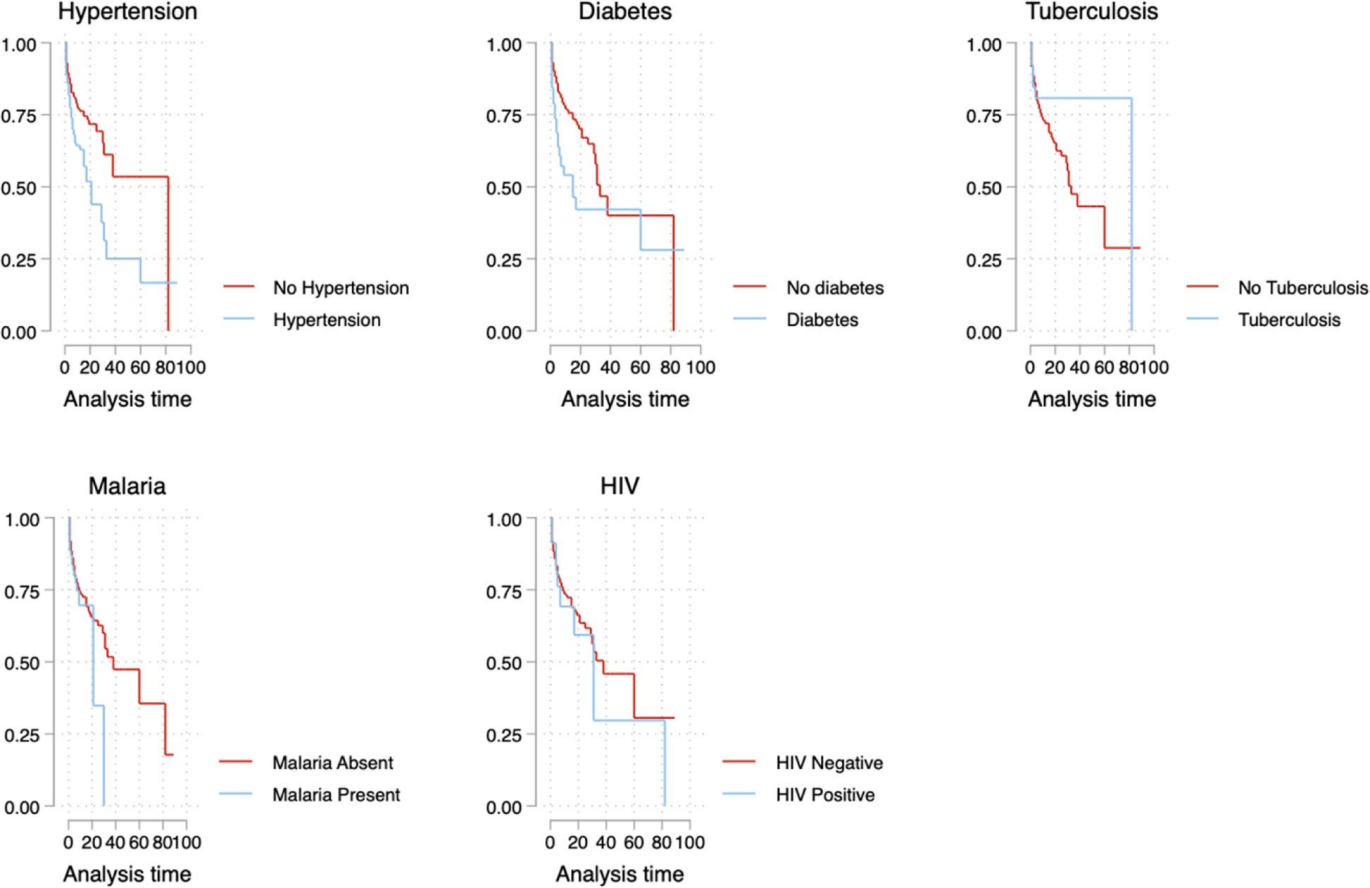
Association between time to death and comorbidities among patients admitted with COVID-19 in selected hospitals in Uganda in 2021

Only 11 participants were vaccinated against COVID-19 (1.1%). Of these, only three participants were fully vaccinated. The most common clinical symptoms experienced by participants were dry cough (587/1026: 57.2%), difficulty in breathing (576/1026: 56.1%), and fever (519/1026: 50.6%) (Fig. 1). A total of 293/1040 (28.2%) were hypertensive, whereas 165/1040 (15.9%) had diabetes mellitus. Malaria was diagnosed among 80/1040 (7.7%). 26/1040 (2.5%) of patients had tuberculosis (Fig. 2). The most common medications used in the management of the patients were dexamethasone, oxygen therapy, azithromycin, zinc, vitamin C and enoxaparin (Fig. 3). Supplementary file 1 (Figs. 4, 5 and 6) shows that most common combination of symptoms were dry cough, difficulty in breathing, and fever (*n* = 46), followed by dry cough and difficulty in breathing (*n* = 45), and dry cough, difficulty in breathing and chest pain (*n* = 38) while the most common combination of medications were zinc, dexamethasone, vitamin C, Azithromycin, Oxygen, Enoxaparin, and Ivermection (*n* = 81), followed by only Zinc (*n* = 44), and Zinc, Vitamin C, and Azithromycin (*n* = 37). Furthermore, the most common comorbidity was hypertension alone (*n* = 181), Hypertension and Diabetes (*n* = 90), and Diabetes Mellitus alone (*n* = 64).

### COVID-19 mortality

A total of 234 out of 1040 (22.5%: 95%CI 12.9 to 36.2%) of patients admitted with COVID-19 in six selected hospitals in Uganda died. The mortality rate was 30.7 deaths per 1000 person days, 95% CI (26.9 to 35.0) (Fig. 4). No patient under 18 years died. The median survival time was 33 days, IQR 9–82. Among patients who died, the median survival time was 3 days, IQR 1–5 (Fig. 5). Sixty-seven patients were transferred out of the hospitals we studied but unfortunately, we could not determine their outcomes.

### Factors associated with the rate of death among COVID-19 patients

Factors associated with time to COVID-19 death included age greater than or equal to 60 compared to those aged less than 60 years Adjusted Hazards Ratio 2.4 [1.7, 3.4], participants with a history of diabetes mellitus had 40% higher hazards as those without a history of diabetes mellitus [1.4 [1.0, 2.2]]. Participants with a positive malaria test at admission had 70% higher hazards than those without a positive malaria test [2.0 [1.0, 3.9]], and a COVID-19 severity score of severe/critical was associated with four times the hazards of death as those with a severity score of mild [6.7 [1.5, 29.1]]. Admission into a public hospital was associated with 48% lower hazards of death compared to those who were admitted in a private hospital or a private not for profit hospital 0.52 [0.35, 0.78] (Table 2, Fig. 5).

**Table 2:** Factors associated with time to COVID-19 death among COVID-19 patients hospitalized in selected hospitals in Uganda in 2021

Variable	cHR [95% CI)	aHR [95% CI]	aTR
**Age**			
Age less than 60 years	1	1	1
Age greater than or equal to 60 years	3.1 [2.3, 4.0]	**2.4 [1.7, 3.4]**	**0.37 [0.24, 0.56]**
**Sex**			
Female	1	1	1
Male	1.1 [0.8, 1.4]	1.1 [0.81, 1.6]	0.98 [0.67, 1.4]
**Type of Hospital**			
Private/PNFP	1	1	1
Public	0.4 [0.3, 0.6]	**0.52 [0.35, 0.78]**	**2.5 [1.6, 3.9]**
**Day of week admitted**			
Workday	1	1	1
Weekend	1.2 [0.9, 1.6]	1.1 [0.75, 1.6]	0.92 [0.60, 1.4]
**COVID-19 Vaccination status**			
Not vaccinated	1	1	1
Vaccinated	1.9 [0.7, 5.0]	1.4 [0.43, 4.5]	0.95 [0.20, 4.5]
**HIV**			
Negative	1	1	1
Positive	1.2 [0.6, 2.3]	1.2 [0.51, 2.6]	1.2 [0.43, 3.1]
**Hypertension**			
Absent	1	1	1
Present	1.8 [1.4, 2.3]	0.93 [0.63, 1.4]	0.96 [0.61, 1.5]
**Diabetes Mellitus**			
Absent	1	1	1
Present	2.1 [1.6, 2.9]	1.4 [0.96, 2.2]	0.64 [0.39, 1.0]
**Tuberculosis**			
No	1	1	1
Yes	0.7 [0.3, 1.7]	2.2 [0.90, 5.3]	0.35 [0.12, 1.0]
**Malaria test**			
Negative	1	1	1
Positive	1.2 [0.8, 2.0]	**2.0 [1.0, 3.9]**	**0.35 [0.16, 0.74]**
**COVID-19 Severity Score**			
Mild	1	1	1
Moderate	1.4 [0.5, 3.9]	1.9 [0.42, 8.4]	0.54 [0.12, 2.4]
Severe/critical	6.0 [2.2, 16.5]	**6.7 [1.5, 29.1]**	**0.13 [0.03, 0.56]**
**Blood pressure stage at admission**			
< 140/< 90	1	1	1
Stage II	0.97 [0.57,1.66]	0.77 [0.44, 1.3]	1.5 [0.78, 2.7]
Stage III/IV	2.0 [0.97,4.1]	1.6 [0.78, 3.4]	0.58 [0.24, 1.4]
**Tachycardia (PR > 100)**			
< =100	1		1
> 100	1.6 [1.2,2.0]	1.1 [0.80, 1.6]	0.76 [0.51, 1.1]

#### Accelerated failure time model

The survival time of patients aged 60 years or more is estimated to be 63% shorter than for patients aged less than 60 years [aTR 0.37, 95% CI 0.24, 0.56]. The survival time of patients admitted in public hospitals was 2.5 times that of patients admitted in private hospitals [aTR 2.5, 95%CI 1.6, 3.9]. Patients with malaria had 65% shorter survival than those without malaria [aTR 0.35, 95%CI 0.16, 0.74]. Finally, patients with a severe or critical COVID-19 severity score had 87% shorter survival than those with a mild score [aTR 0.13, 95% CI 0.03, 0.56] (Table 2).

## Discussion

This aimed at describing in hospital mortality and associated factors among COVID-19 patients admitted at six health facilities in Uganda between January 2021 and December 2021. In hospital mortality among COVID-19 patients was high (22.5%: 95%CI 12.9 to 36.2%). The high mortality could be explained by the emergence of delta SARS CoV-2 variant in Uganda that is believed to be more pathogenic, highly transmissible, associated with severe disease and reduced diagnostic sensitivity [27, 28]. The high in hospital mortality may also be linked to limited ICU space, mechanical ventilators and skilled healthcare workers such as specialized ICU nurses and intensivists to manage severe and critical COVID-19 cases [17].

The in hospital mortality in this study was higher than that in Ethiopia (11.1%) [29], India (8.1%) [30], New York, and USA (13.1%) [31]. However, the in-hospital mortality in this study was similar to that from Italy (25.2%) [9], and Belgium (29.9%) [32]. These differences could be due to variations in the pathogenicity, transmissibility of the SARS CoV-2 variants in the different countries and also differences in the capacity of the health systems to manage COVID-19 cases [33, 34]. Nevertheless, the in hospital mortality in this study is lower than that reported from previous studies done in Uganda; at a national referral COVID-19 Treatment Unit (37%) [14] and Kampala metropolitan area (67%) [17]. It is also lower than that reported from studies done in Somalia (40%) [35], in DRC (32%) [20] and in South Africa (43%) [18]. The high in hospital mortality in these studies as compared to our finding may be due to differences in the study period considered.

The median survival time was 33 days, IQR 9 to 82. Among patients who died, median survival time was 3 days IQR, 1 to 5. The previous studies done on in hospital mortality in Uganda did not estimate median time to death [14, 17]. Our finding differ from studies conducted in the USA (6 days) [13], Ethiopia (9 days) [29], Mexico (19 days) [11] and China (18.5 days) [8]. This might be due to differences in the virulence and pathogenicity of the SARS CoV-2 variants in the different countries and also health system’s capacity to manage COVID-19 [33, 34].

This study revealed that older age (greater than 60 years) was associated with mortality among COVID-19 patients, a finding that is consistent with studies that also reported that old age has an increased risk of dying from COVID-19 [8, 10–12, 14, 16, 20, 35]. This could be attributed to the fact that the body’s immune system particularly the cell mediated immunity deteriorates with age and also the presence of comorbidities that make older persons more prone to severe COVID-19 disease and eventually death [36].

An increased risk of mortality among patients with diabetes mellitus was also showed. In line with this finding, high risk of mortality among patients with diabetes mellitus has also been reported in other studies conducted in China, Italy and New York [8, 9, 13, 19]. This could be related to the fact that diabetes mellitus weakens both the innate and adaptive immune system thus patients with diabetes mellitus are prone to severe COVID-19 illness and death [37]. Furthermore, Diabetes mellitus is associated with elevated cardiac troponin levels [38, 39], a study conducted in Italy to determine the prevalence and prognostic value of cardiac troponin in elderly patients found out that increased cardiac troponin levels in COVID-19 is a risk factor of in hospital mortality [40].

Patients with a COVID-19 severity score of severe/critical had an increased risk of death compared to those with a COVID-19 severity score of mild. COVID-19 severity scores have been identified as reliable tools in the prediction of in hospital mortality among COVID-19 patients [41]. The COVID-19 severity score reflects the patient’s state and wellbeing in relation to the disease through assessment of the patient using parameters such as age, respiratory rate, heart rate, oxygen saturation, consciousness and blood pressure [41]. A COVID-19 severity score of severe/ critical reflects severe/critical COVID-19 illness which has been found to result in high in hospital mortality [42].

Patients with a positive malaria test at admission had a higher risk of mortality as compared to those with a negative test. Similarly a study conducted in Sudan to evaluate the clinical outcomes of patients with concurrent COVID-19 and malaria infection showed that patients with concurrent malaria and COVID-19 had a greater mortality risk [43]. This may be due to the fact malaria and COVID-19 have similar presentations and in the COVID-19 era it may be late diagnosed thus becoming severe, causing organ failure and subsequently death [44]. Additionally, malaria parasitemia is associated with high ferritin levels [45], a study conducted in four Italian centers in Italy to evaluate the prognostic role of ferritin in COVID-19 patients showed that high ferritin levels are associated with poor COVID-19 outcomes [46]. Admission to a public hospital was associated with low risks of mortality. This could be attributed to the fact that most of patients that sought medical care from private for profit or private not for profit hospitals were severe/critical Covid-19 cases probably due to limited ICU bed capacity in public hospitals and for better clinical care. Public hospitals also occasionally referred their severe/ critical cases to private for profit or private not for profit hospitals because of lack of enough ICU bed capacity to accommodate all severe cases. Severe and critical cases of COVID-19 were associated with high levels of in hospital mortality [42].

### Strengths and limitations

One of the strengths of this study is that it was a multi-center study including public-private mix of hospitals in both urban and rural settings thus our results are representative of a bigger population of hospitalized COVID-19 patients in the country. The sample size was powered enough to identify potential associations between the variables of interest. However, due to the retrospective nature of the study, obtaining all the data in relation to parameters of interest was not possible. Additionally, missing information in some files was also a challenge.

The outcome was defined as all-cause mortality because autopsies were not routinely done to ascertain the cause of death. Also, a challenge of confirming whether patients were sick or admitted because of COVID-19 or with COVID-19 was faced.

Finally, ascertaining the outcomes of the participants who were transferred out of the participating hospitals was not possible, which could potentially understate our mortality estimates.

## Conclusions

In hospital mortality among COVID-19 patients was high. Factors associated with shorter time to death included; Age ≥ 60 years, history of diabetes mellitus, having malaria at admission. Admission in a public hospital was associated with longer survival. We therefore recommend close monitoring of COVID-19 patients that are elderly and those with comorbidities. We also recommend screening for malaria among all patients admitted with COVID-19.

## Supplementary Information

**Additional file 1: Figure 4.** Intersection of symptoms experienced by COVID-19 patients hospitalized in selected hospitals in Uganda in 2021. **Figure 5.** Intersection of comorbidities of COVID-19 patients hospitalized in selected hospitals in Uganda in 2021. **Figure 6.** Intersection of medications given to COVID-19 patients hospitalized in selected hospitals in Uganda in 2021.

## Data Availability

The datasets used and/or analysed during the current study are available from the corresponding author on reasonable request.

## Abbreviations

COVID-19: Coronavirus disease 2019
AIDS: Acquired Immunodeficiency Syndrome
HIV: Human Immunodeficiency Virus
WHO: World Health Organization
PCR: Polymerase chain reaction
RDT: Rapid diagnostic test
IQR: Interquartile range
AFT: Accelerated failure time
