# Supplementary material for: Survival analysis of patients with COVID-19 admitted at six hospitals in Uganda in 2021: a cohort study
Source: Arch Public Health. 2022 Nov 15;80:233. doi: 10.1186/s13690-022-00991-3 (PMC9666944; doi:10.1186/s13690-022-00991-3)
Supplement: Supplementary file 1 — Additional file 1: Figure 4. Intersection of symptoms experienced by COVID-19 patients hospitalized in selected hospitals in Uganda in 2021. Figure 5. Intersection of comorbidities of COVID-19 patients hospitalized in selected hospitals in Uganda in 2021. Figure 6. Intersection of medications given to COVID-19 patients hospitalized in selected hospitals in Uganda in 2021. [file 13690_2022_991_MOESM1_ESM.docx]

**
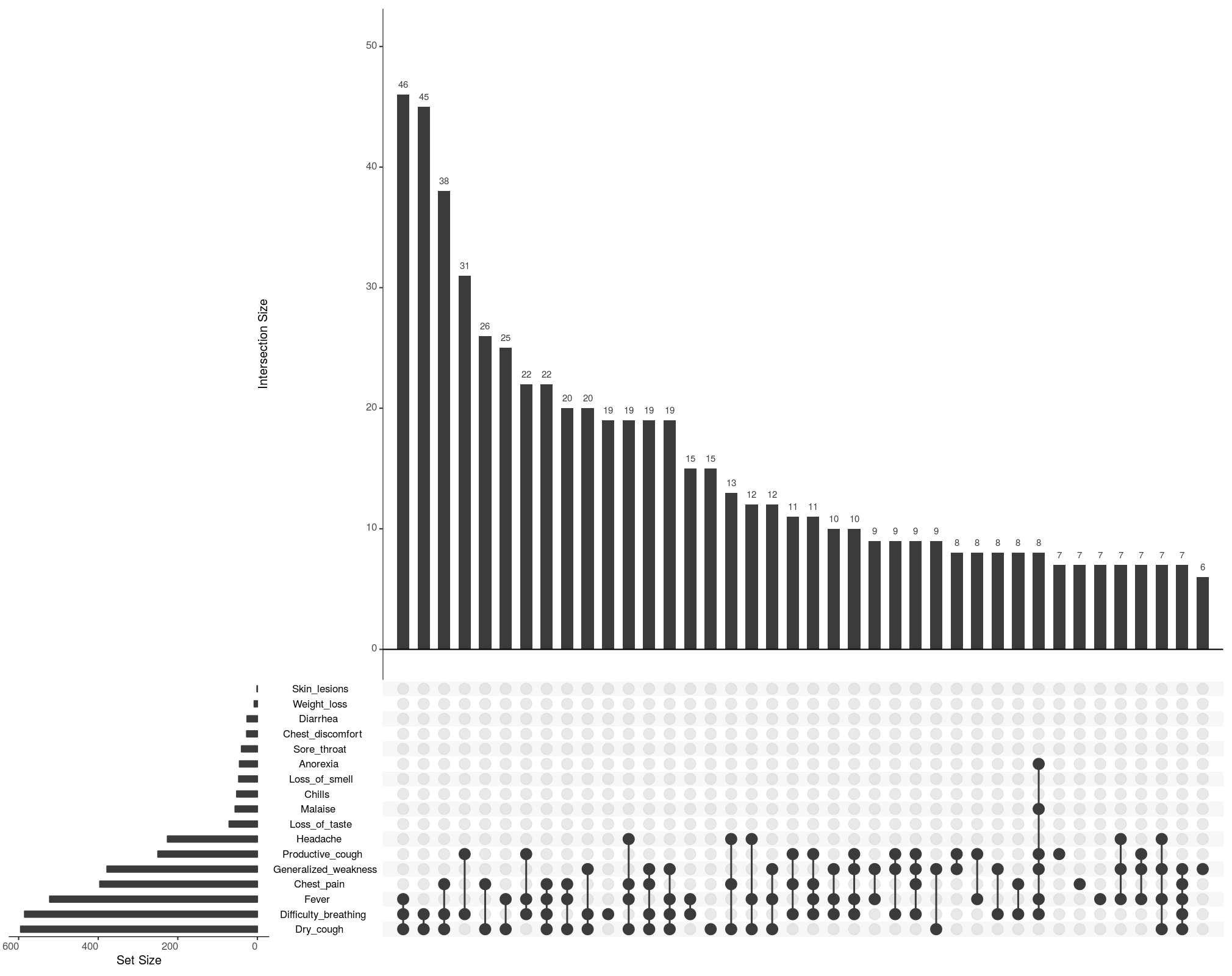
**

Figure 4: Intersection of symptoms experienced by COVID-19 patients hospitalized in selected hospitals in Uganda in 2021

**
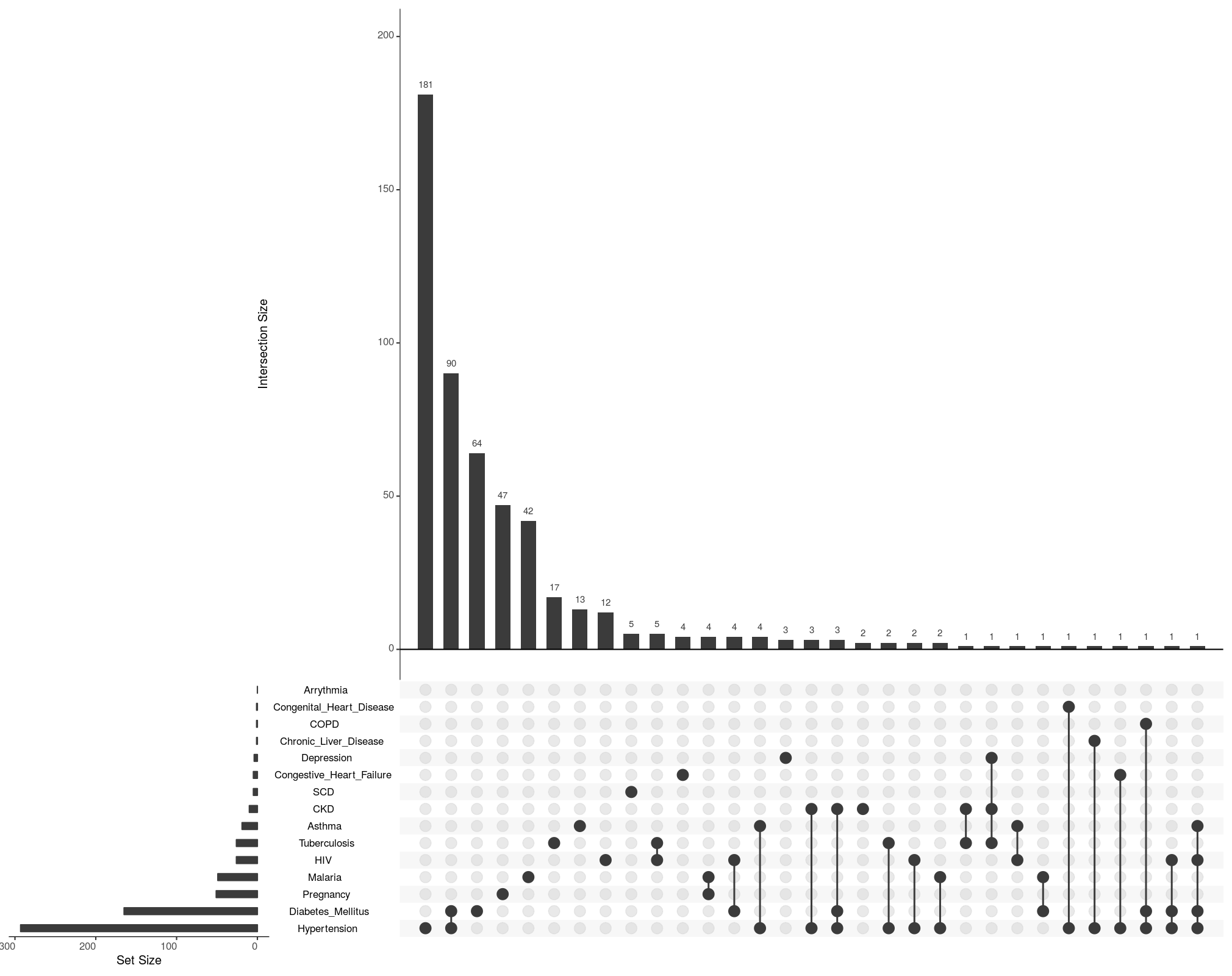
**

Figure 5: Intersection of comorbidities of COVID-19 patients hospitalized in selected hospitals in Uganda in 2021


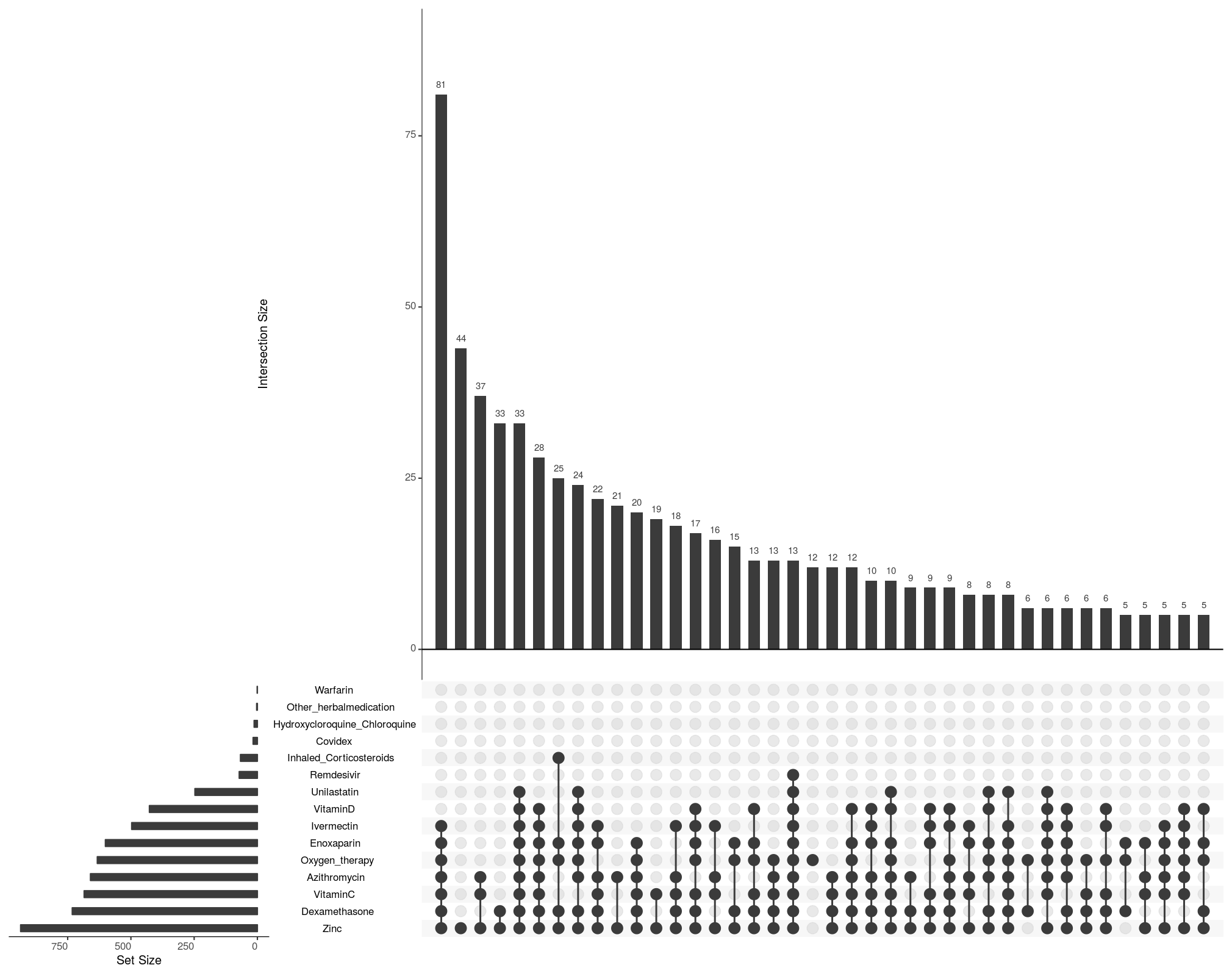


Figure 6: Intersection of medications given to COVID-19 patients hospitalized in selected hospitals in Uganda in 2021
